# THEORETICAL ANALYSIS OF THE DISABLEMENT PROCESS MODEL IN THE CONTEXT OF VISION LOSS IN LATE LIFE

**DOI:** 10.1093/geroni/igae098.3542

**Published:** 2024-12-31

**Authors:** Haruno Suzuki, Margaret Wallhagen

**Affiliations:** University of California San Francisco, San Francisco, California, United States; University of California San Francisco, San Francisco, California, United States

## Abstract

Vision loss experienced by older adults is associated with poor biopsychosocial health, yet the mechanisms underpinning adjustment to this disability are poorly understood. One framework that may facilitate the exploration of the adjustment is the disablement process model (DPM) (Verbrugge & Jette, 1994), which describes how a chronic condition leads to disability. However, limited studies have evaluated the model’s applicability to older adults living with vision loss. This study critically appraises the theoretical assumptions of DPM for its applicability to studying vision loss acquired in late life. We utilized the criteria adapted from Chinn and Kramer (2011), which include 6-items of description of theory and 5-items of critical reflection. The central pathway of the DPM describes moving from pathology to impairments, functional limitations, and disability with intra- and extra-individual factors influencing the experience of disability. Definitions of each concept and subconcept are explicit yet general enough to use in various populations with disabilities, increasing the model’s generalizability. However, the magnitude of the effects of each subconcept on disability and health outcomes is unclear. Furthermore, it is based on a medical model of disability, focusing on preventing disability. It lacks the nuance of social models that include strengths and capacities, thus limiting its use in developing inclusive communities that reinforce intrinsic capability and extrinsic environmental supports, such as age-friendly community. The findings imply the necessity of modifying the model to improve its utility in exploring the lived experience of disability among older adults living with vision loss in late life.

